# Mapping global trends in adipose-derived mesenchymal stem cell research: A bibliometric analysis using scopus database

**DOI:** 10.1016/j.amsu.2022.103542

**Published:** 2022-04-14

**Authors:** Khan Sharun, Taha Hussein Musa, Hassan Hussein Musa, Rohit Kumar, A.M. Pawde, Vikash Chandra, Hardeep Singh Tuli, Kuldeep Dhama, Amarpal, G. Taru Sharma

**Affiliations:** a Division of Surgery, ICAR-Indian Veterinary Research Institute, Izatnagar, Bareilly, Uttar Pradesh, India; b Biomedical Research Institute, Darfur University College, Nyala, Sudan; c Key Laboratory of Environmental Medicine Engineering, Ministry of Education, Department of Epidemiology and Health Statistics, School of Public Health, Southeast University, Nanjing, Jiangsu Province, China; d Faculty of Medical Laboratory Science, University of Khartoum, Khartoum, Sudan; e Division of Physiology and Climatology, ICAR-Indian Veterinary Research Institute, Izatnagar, Bareilly, Uttar Pradesh, India; f Department of Biotechnology, Maharishi Markandeshwar (Deemed to be University), Mullana, Ambala, 133207, Haryana, India; g Division of Pathology, ICAR-Indian Veterinary Research Institute, Izatnagar, Bareilly, Uttar Pradesh, India; h National Institute of Animal Biotechnology, Hyderabad, 500032, India

**Keywords:** Mesenchymal stem cells, Adipose-derived mesenchymal stem cell, Adipose tissue-derived mesenchymal stem cell, Bibliometric analysis, Research progress, Research trends, Scopus, MSC, Mesenchymal stem cell, AdMSC, Adipose-derived mesenchymal stem cells, WoS, Web of Science

## Abstract

**Background and objective:**

Adipose-derived mesenchymal stem cells (AdMSC) are multipotent adult mesenchymal cells isolated and cultured from the stromal vascular fraction derived from adipose tissue. The present study was conducted to analyze the global trends in AdMSC research using bibliometric and visual analysis tools.

**Methods:**

The literature search was done on February 13, 2022, using appropriate keywords and inclusion-exclusion criteria from the Scopus database. The extracted data were retrospectively analyzed and visualized using Bibliometrics and R packages and VOSviewer.

**Results:**

Preliminary analysis identified 1569 documents from the Scopus database published between 2005 and 2021. The average citations received per document was 26.51, whereas the average citations per year per document was 3.347. In addition, the selected documents had an *h*-index value of 90. China was the most productive country, whereas Seoul National University (South Korea) was identified as the most productive institute/university in AdMSC research. In addition, the National Natural Science Foundation of China funded the most research studies in AdMSC research.

**Conclusion:**

The findings from this study indicate a progressive increase in interest among the research community towards AdMSC, suggesting promising prospects in the coming years.

## 1 Introduction

Stem cells play a major role in different cell-based and cell-free therapeutic strategies [[1], [2], [3]]. They are isolated and cultured from several sources. Bone marrow and adipose tissue are the two important sources of mesenchymal stem cells (MSC) [4]. Adipose-derived mesenchymal stem cells (AdMSC) are multipotent adult mesenchymal cells isolated and cultured from the stromal vascular fraction derived from adipose tissue [5]. They can differentiate into several cell lineages such as adipocytes, chondrocytes, cardiomyocytes, hepatocytes, osteoblasts, vascular endothelial cells, pancreatic cells, and neural cells [6]. AdMSC is gaining importance in regenerative medicine due to the higher yield of MSCs (100–1000 times) compared to bone marrow-derived mesenchymal stem cells (BM-MSC) [7]. In addition, adipose tissue can be harvested easily by minimally invasive surgical techniques, processed by enzymatic or non-enzymatic methods, and isolated and cultured to obtain AdMSC [5,8].

AdMSC has therapeutic applications in musculoskeletal pathologies such as osteochondral focal defects, knee, and hip osteoarthritis, rotator cuff, and Achilles tendinopathies [8]. In addition, they are also evaluated for utility in wound repair, renal repair, hepatic repair, myocardial repair, neuroprotection, neurotrophic effects, and other regenerative medicine applications [9]. Over the past decade, there has been a rapid increase in the publications as well as several novel concepts and advancements in the field of AdMSC research [10]. Therefore, a systematic assessment of research has to be conducted to evaluate the progress of science towards research on AdMSC.

Bibliometric analysis is a systematic method used to collect and analyze large volumes of scientific data. It helps to identify the ongoing trends in a specific field while providing a glance at the emerging areas in that field [11]. In addition, the bibliometric analysis also helps to understand the emerging trends in publication patterns, journal performance, and collaboration patterns [11]. Scopus is the multidisciplinary database developed by Elsevier and first launched in November 2004 [12]. It is an ideal database that is better suited for analyzing research results. In addition, Scopus has more inclusive content coverage than Web of Science (Clarivate), making it more convenient for bibliometric analysis [12].

The present study was conducted to analyze the global trends in AdMSC research within the Scopus database using bibliometric and visualization tools. Furthermore, the study was designed in such a way as to gain a better understanding of the present scenario in stem cell research by analyzing different characteristics.

## 2 Materials and methods

### 2.1 Search strategy and data collection

The literature search and data collection was done on February 13, 2022, from the Scopus database (available at: https://www.scopus.com/home.uri). The search was limited to journal articles published in the English language till 2021. Therefore, we excluded all publications such as reviews, editorials, book chapters, books, conference papers, letters, notes, short surveys, erratum, reports, and retracted papers. In addition, all journal articles published in other languages were also excluded. The following search strategy was used for data collection from the Scopus database:

TITLE (“adipose-derived mesenchymal stem cell” OR “adipose derived mesenchymal stem cell” OR “adipose tissue-derived mesenchymal stem cell” OR “adipose tissue derived mesenchymal stem cell”) AND (LIMIT-TO (SRCTYPE, “j")) AND (LIMIT-TO (DOCTYPE, “ar”)) AND (LIMIT-TO (LANGUAGE, “English”)) AND (EXCLUDE (PUBYEAR, 2022)).

### 2.2 Analysis of variables

The data was extracted from the selected articles in terms of the following variables: articles published each year, top ten authors, institutes, countries, and journals having the highest number of published articles, top ten funding agencies, and top ten most cited articles related to AdMSC research. In addition, the 2021 Journal Impact Factor™ (JIF) of the top ten journals was extracted from the Journal Citation Reports™ (JCR). The metadata was analyzed using the R studio and bibliometrix package for the R statistical programming language as a unique open-source tool designed for performing comprehensive science mapping analysis [13].

### 2.3 Visualization

The extracted data were visualized using the network visualization software VOSviewer (https://www.vosviewer.com) [14]. The software was used for the graphical representation of bibliometric maps.

## 3 Results

### 3.1 Scopus database output

**Fig. 1:**
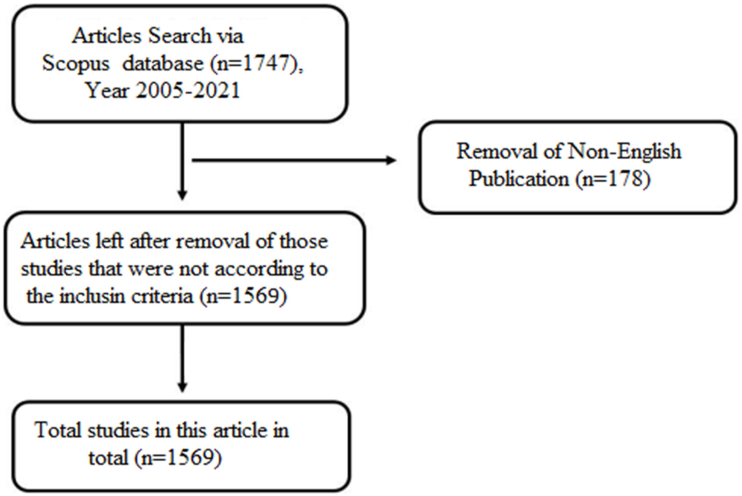
Summary of the search outcome indicating the number of articles in adipose-derived mesenchymal stem cell research retrieved from Scopus database.

**Fig. 2:**
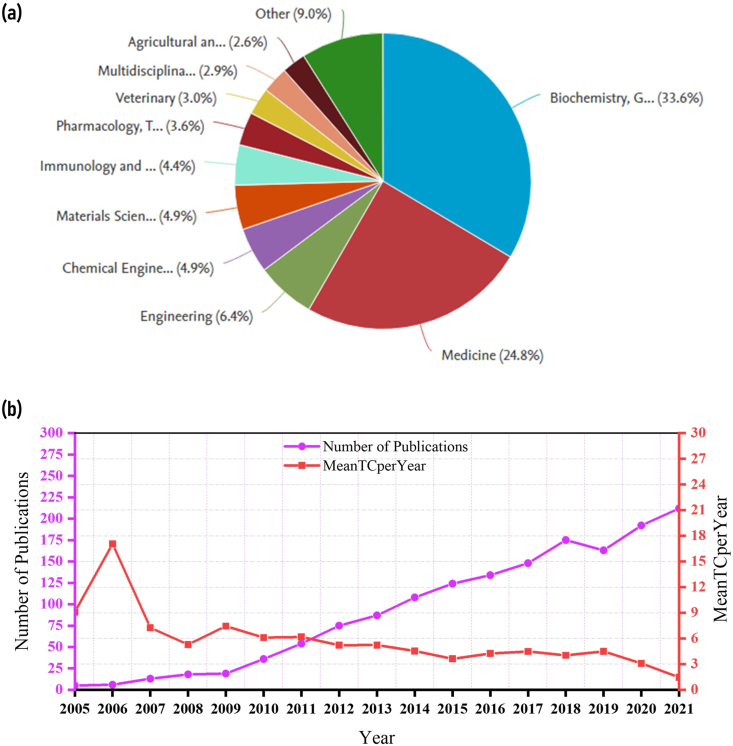
**(a)** Pie chart illustrating the top ten subject areas under which articles on adipose-derived mesenchymal stem cell research are classified in the Scopus database. **(b)** Annual distribution of total publications and Average Mean of Total Citation Per Year (MeanTCperYear) during 2005–2021 on adipose-derived mesenchymal stem cell research.

The initial search identified 1747 documents in the Scopus database published between 2005 and 2021. However, after refining the search protocol based on inclusion and exclusion criteria, 1569 articles from 618 sources were selected for further analysis and data extraction (Fig. 1). The Scopus database categorized these 1569 articles into 25 subject areas. Among these, the top 10 subject areas in AdMSC research are biochemistry, genetics and molecular biology 906 (33.6%), medicine 669 (24.8%), engineering 174 (6.4%), chemical engineering 133 (4.9%), materials science 131 (4.9%), immunology and microbiology 119 (4.4%), pharmacology, toxicology and pharmaceutics 98 (3.6%), veterinary 80 (3.0%), and multidisciplinary 78 (2.9%) (Fig. 2a). The average citations received per document were 26.51, whereas the average citations per year per document were 3.347 (Table 1). In addition, the articles had an *h*-index value of 90. The *h*-index is calculated based on the highest number of papers included that have had at least the same number of citations.

**Table 1:** Basic characteristics of the study sample.

Description	Results	Description	Results
Timespan	2005:2021	**Authors**	
Sources (Journals, Books, etc)	618	Authors	7056
Documents	1569	Author Appearances	11510
Affiliation	160	Authors of single-authored documents	2
Funding Sponsor	159	Authors of multi-authored documents	7054
Average years from publication	5.41	**Authors Collaboration**	
Average citations per documents	26.51	Single-authored documents	2
Average citations per year per doc	3.347	Documents per Author	0.222
References	60938	Authors per Document	4.5
**Document Types**		Co-Authors per Documents	7.34
Article	1569	Collaboration Index	4.5
**Document Contents**			
Keywords Plus (ID)	9996		
Author's Keywords (DE)	3007		

### 3.2 Time trend of publications

The annual global trend in total publications is shown in Fig. 2b. A steady increase in the publications was observed between 2005 and 2021, with a peak in 2021. This indicates that AdMSC research is rapidly gaining importance in the research community. Among the 1569 articles extracted from the Scopus database, 212 were published in 2021. The total citations received increased from 2005 to 2021. In addition, the average mean of total citation per year (MeanTCperYear) during the year 2005–2021 on adipose-derived mesenchymal stem cell research is plotted in Fig. 2b.

### 3.3 Most productive institutes and countries involved in AdMSC research

The top ten productive institutes/universities and countries publishing (based on total publications) on AdMSC research were identified. Among the 1569 articles evaluated, the corresponding authors of 410 publications originated from China, followed by South Korea (232) and Iran (141) (Table 2). Other productive countries included the United States (104), Japan (77), Spain (57), Italy (55), Germany (47), Turkey (40), and Brazil (34). Among the countries, China has a robust national collaboration in AdMSC research with 361 single-country publications (indicative of intra-country collaboration) and 49 multiple-country publications (indicative of inter-country collaboration). However, the top ten listed countries involved in AdMSC research have a low Multiple Country Publications ratio (MCP Ratio less than 0.50), indicating lower international collaboration.

**Table 2:** Top ten corresponding author's countries and most cited countries publishing on adipose-derived mesenchymal stem cell research.

Corresponding Author's Country						Most Cited Countries			Country Scientific Production	
Country	Articles	Freq	SCP	MCP	MCP Ratio	Country	TNC	AAC	Region	Freq
China	410	0.28	361	49	0.1195	China	10215	24.91	China	1477
South Korea	232	0.16	206	26	0.1121	South Korea	8087	34.86	South Korea	751
Iran	141	0.10	126	15	0.1064	Spain	3868	67.86	Iran	655
USA	104	0.07	72	32	0.3077	USA	3516	33.81	USA	423
Japan	77	0.05	71	6	0.0779	Italy	2170	39.45	Japan	297
Spain	57	0.04	41	16	0.2807	Japan	2073	26.92	Spain	255
Italy	55	0.04	44	11	0.2000	Iran	1747	12.39	Italy	224
Germany	47	0.03	27	20	0.4255	Netherlands	1327	73.72	Turkey	169
Turkey	40	0.03	38	2	0.0500	Germany	1277	27.17	Germany	148
Brazil	34	0.02	31	3	0.0882	Switzerland	537	107.40	Brazil	134

SCP: Single Country Publication (intra-country collaboration); MCP: Multiple Country Publications (inter-country collaboration); TNC: Total Number of Citations; AAC: Average Article Citations.

**Fig. 3:**
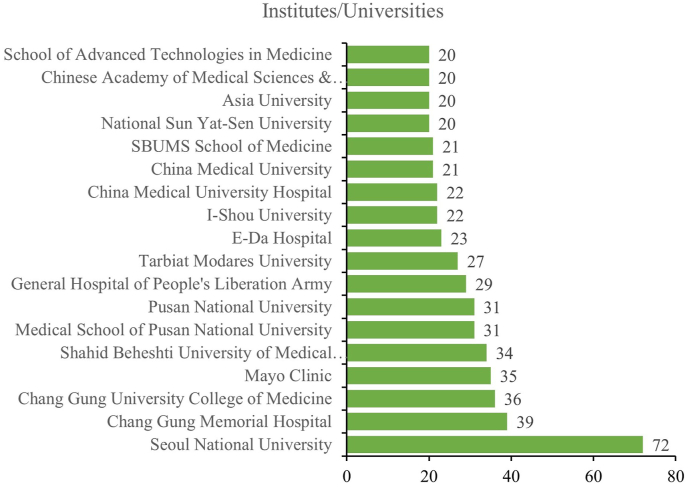
The top 18 institutes/universities of 160 reported contributed to adipose-derived mesenchymal stem cell research from 2005 to 2021.

Similarly, 72 articles originated from the Seoul National University, followed by Chang Gung Memorial Hospital (39) and Chang Gung University College of Medicine (36). Other productive institutes/universities included Mayo Clinic (35), Shahid Beheshti University of Medical Sciences (34), Medical School of Pusan National University (31), Pusan National University (31), General Hospital of People's Liberation Army (29), Tarbiat Modares University (27), E-Da Hospital (23), I-Shou University (22), China Medical University Hospital (22), China Medical University (21), SBUMS School of Medicine (21), National Sun Yat-Sen University (20), and Asia University (20) (Fig. 3).

### 3.4 Top ten authors involved in AdMSC research

Among the 7056 authors identified, the characteristics of the top ten productive individuals are given in Table 3. These prolific authors included Wang Y. (50, 3.19% of 1569 articles), Yip H.K. (34, 2.17%), Zhang Y. (30, 1.921%), Youn H.Y. (26, 1.66%), Li Q. (23, 1.47%), Li J. (22, 1.40%). Liu Y. (22, 1.40%), Chen YL (21, 1.34%), Li H (21, 1.34%), and Kim JH (20, 1.27%).

**Table 3:** The characteristics of top ten most productive authors over time publishing on adipose-derived mesenchymal stem cell research.

Element^a^	h_index	g_index	TNC	TNP
Wang Y	20	30	1054	50
Yip HK	21	34	1756	34
Zhang Y	13	26	686	30
Youn HY	12	21	471	26
Li Q	13	20	436	23
Li J	15	22	929	22
Liu Y	15	22	720	22
Chen YL	14	21	1073	21
Li H	14	21	848	21
Kim JH	14	20	836	20

TNC: Total Number of Citations, TNP: Total Number of publications.

a Ranking based on total publications (data collected till February 13, 2022).

### 3.5 Top ten journals publishing AdMSC research

**Fig. 4:**
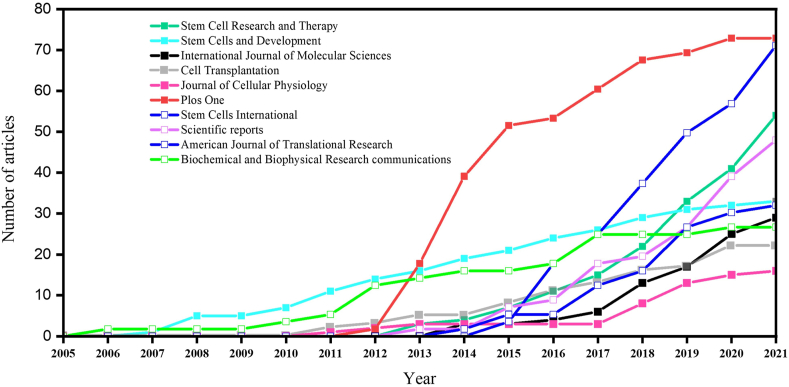
The publication trend of top ten journals publishing on adipose-derived mesenchymal stem cell research from 2005 to 2021.

Among the 1569 articles evaluated in this study, 50 articles were published in Stem Cell Research and Therapy (3.19%), followed by 40 articles in Plos One (2.55%), and 33 articles in Stem Cells and Development (2.10%). The other important journals include Stem Cells International (33, 2.10%), International Journal of Molecular Sciences (26, 1.66%), Scientific Reports (25, 1.59%), Cell Transplantation (22, 1.40%), American Journal of Translational Research (18, 1.15%), Journal Of Cellular Physiology (16, 1.02%), and Biochemical and Biophysical Research Communications (15, 0.96%) (Table 4). The publication pattern of the top ten journals is illustrated in Fig. 4. Among the top ten journals, Stem Cell Research and Therapy journal has the highest Journal Impact Factor™ (Journal Citation Reports - Clarivate Analytics).

**Table 4:** The characteristics of top ten journals publishing on adipose-derived mesenchymal stem cell research.

Element^a^	h_index	TNC	TNP	JIF™ (2021)^b^	CiteScore (2020)^c^	Publisher
Stem Cell Research and Therapy	19	1245	50	6.832	7.9	Springer Nature
Plos One	23	1426	40	3.240	5.3	Public Library of Science
Stem Cells and Development	20	2035	33	3.272	5.9	Mary Ann Liebert
Stem Cells International	14	591	33	5.443	7.2	Hindawi
International Journal of molecular Sciences	14	360	26	5.923	6.0	Multidisciplinary Digital Publishing Institute (MDPI)
Scientific reports	9	693	25	4.379	7.1	Springer Nature
Cell Transplantation	12	576	22	4.064	5.6	SAGE
American Journal of Translational Research	10	293	18	4.060	5.3	e-Century Publishing Corporation
Journal of Cellular Physiology	11	438	16	6.384	8.9	Wiley-Blackwell
Biochemical and Biophysical Research Communications	12	696	15	3.575	5.5	Academic Press Inc Elsevier Science

TNC: Total Number of Citations; TNP: Total Number of Publications.

a Ranking based on total publications (data collected till February 13, 2022).

b Retrieved from 2020 Journal Citation Reports (Clarivate Analytics).

c Retrieved from Scopus database (calculated on May 5, 2021).

### 3.6 Top ten most cited articles in AdMSC research

The characteristics of the top ten most cited articles on AdMSC research are given in Table 5. None of the journals that published the top ten articles have more than one article. Furthermore, the table was topped by the article published in the journal Stem Cells (Oxford University Press), followed by The Lancet (Elsevier), Gastroenterology (AGA Institute), Hepatology (American Association for the Study of Liver Diseases), Osteoarthritis and Cartilage (Osteoarthritis Research Society International), Journal of the American Chemical Society (American Chemical Society), Stem Cells and Development (International Federation of Adipose Therapeutics and Science), Arthritis and Rheumatism (American College of Rheumatology), Biochemical and Biophysical Research Communications (Elsevier), and Cytotherapy (International Society for Cell & Gene Therapy).

**Table 5:** The characteristics of top ten most cited articles on adipose-derived mesenchymal stem cell research.

Author, Year, Journal	Article title	TNC	TC per year	Normalized TC
Yañez R. et al., 2006, Stem Cells	Adipose tissue-derived mesenchymal stem cells have in vivo immunosuppressive properties applicable for the control of the graft-versus-host disease	544	32.0000	1.9927
Panés J. et al., 2016, Lancet	Expanded allogeneic adipose-derived mesenchymal stem cells (Cx601) for complex perianal fistulas in Crohn's disease: a phase 3 randomised, double-blind controlled trial	477	68.1429	18.7663
González M.A. et al., 2009, Gastroenterology	Adipose-derived mesenchymal stem cells alleviate experimental colitis by inhibiting inflammatory and autoimmune responses	476	34.0000	4.9206
Banas A. et al., 2007, Hepatology	Adipose tissue-derived mesenchymal stem cells as a source of human hepatocytes	444	27.7500	4.0792
Im G.I. et al., 2005, Osteoarthritis and cartilage	Do adipose tissue-derived mesenchymal stem cells have the same osteogenic and chondrogenic potential as bone marrow-derived cells?	437	24.2778	2.8266
Kim T. et al., 2011, Journal of the American Chemical Society	Mesoporous silica-coated hollow manganese oxide nanoparticles as positive T 1 contrast agents for labeling and MRI tracking of adipose-derived mesenchymal stem cells	429	35.7500	6.3174
Ra J.C. et al., 2011, Stem Cells and Development	Safety of intravenous infusion of human adipose tissue-derived mesenchymal stem cells in animals and humans	402	33.5000	5.9198
González M.A. et al., 2009, Arthritis and Rheumatism	Treatment of experimental arthritis by inducing immune tolerance with human adipose-derived mesenchymal stem cells	396	28.2857	4.0936
Timper K. et al., 2006, Biochemical and Biophysical Research Communications	Human adipose tissue-derived mesenchymal stem cells differentiate into insulin, somatostatin, and glucagon expressing cells	380	22.3529	1.3919
Oedayrajsingh-Varma, M.J. et al., 2006, Cytotherapy	Adipose tissue-derived mesenchymal stem cell yield and growth characteristics are affected by the tissue-harvesting procedure	339	19.9412	1.2418

TNC: Total Number of citations.

^a^Ranking based on total citations (data collected till February 13, 2022).

In addition, the oldest article within the top ten most-cited article was published in the year 2005, while the most recent one was in 2016. The top ten publications contributed 4324 citations to the total citation count. The top-cited article on AdMSC research was published in Stem Cells journal titled “Adipose tissue-derived mesenchymal stem cells have in vivo immunosuppressive properties applicable for the control of the graft-versus-host disease.” The study evaluated the immunoregulatory properties of human and mouse-derived AdMSC. It provided the first experimental proof that AdMSC can control graft-versus-host disease associated with allogeneic hematopoietic transplantation [15].

### 3.7 Top ten funding agencies of AdMSC research

**Fig. 5:**
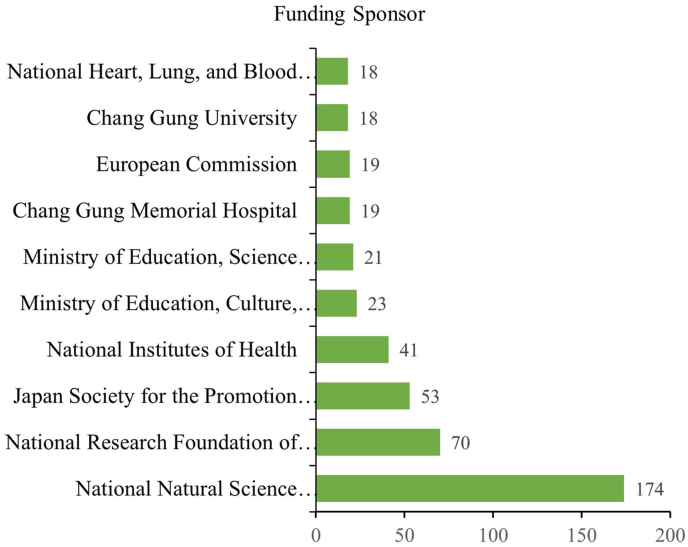
The top 10 funding sponsors of the 159 reported contributed to adipose-derived mesenchymal stem cell research from 2005 to 2021.

Among the research agencies, the National Natural Science Foundation of China funded the most research studies (174) in AdMSC research, followed by the National Research Foundation of Korea (70), Japan Society for the Promotion of Science (53), National Institutes of Health (41), Ministry of Education, Culture, Sports, Science and Technology (23), Ministry of Education, Science and Technology (21), Chang Gung Memorial Hospital (19), European Commission (19), Chang Gung University (18), National Heart, Lung, and Blood Institute (18), Korea Health Industry Development Institute (17), and Ministry of Science, ICT, and Future Planning (16) (Fig. 5).

### 3.8 Network visualization map

**Fig. 6:**
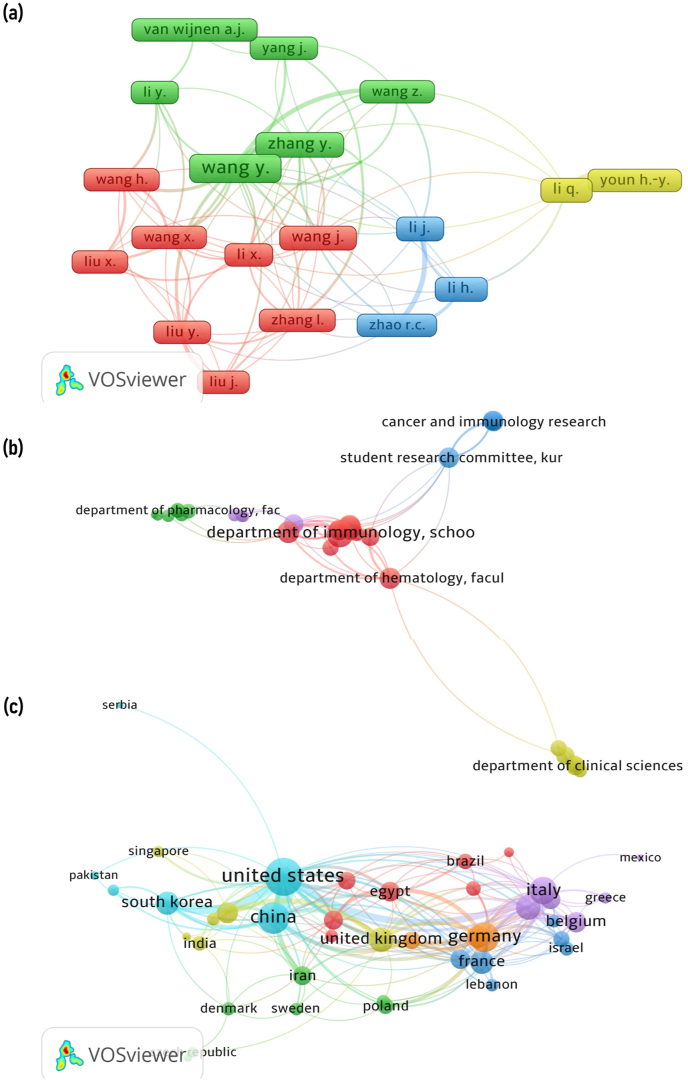
The network visualization map illustrating the relationship between authors with a minimum of 15 documents **(a)**, organizations with a minimum of three documents **(b)**, and countries with a minimum of five documents **(c)**, were selected based on the links and total link strength. The size of the circle indicates the total published articles (larger the circle, more the published articles). In contrast, line width indicates the link strength (wider line indicates more link strength). The distance between two circles indicates the relatedness of the nodes.

Co-author analysis helps to establish the relationship between authors, organizations, and countries based on total link strength. The network indicating the relationship between authors, organizations, and countries is visualized and presented in Fig. 6. The network visualization map illustrating the relationship between authors was plotted among individuals with a minimum of 15 documents. Similarly, organizations with a minimum of three documents and countries with a minimum of five were selected based on the links and total link strength. The size of the circle indicates the total published articles (larger the circle, more the published articles). In contrast, line width indicates the link strength (wider line indicates more link strength). The distance between two circles indicates the relatedness of the nodes.

### 3.9 The conceptual structure of keywords analysis

**Fig. 7:**
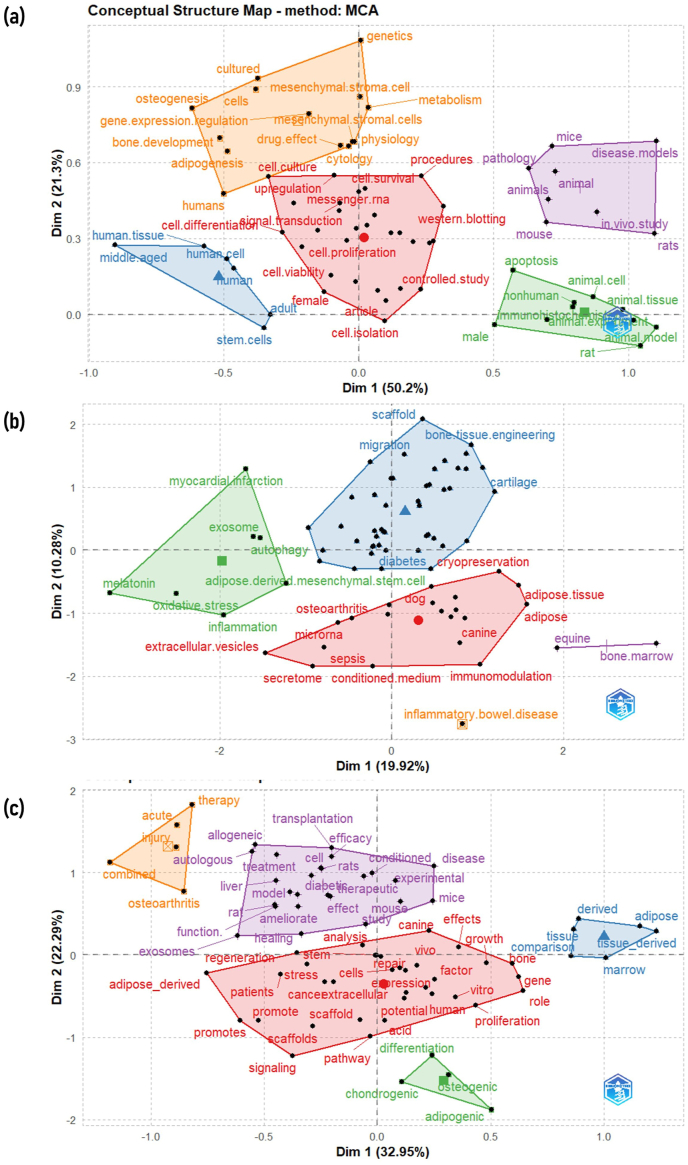
Conceptual structure analysis of the top 75 keywords Plus **(a)**, author keywords **(b)**, and keywords in titles **(c)**, using Multiple Correspondence Analysis (MCA).

The analysis of the 75 keywords plus found to be distributed into five clusters as **Cluster 1** (mice, pathology, animals, mouse, rats, in vivo study, disease. models, and animal), **Cluster 2** (apoptosis, animal. cell, animal. tissue, animal model, rat, animal. experiment, immunohistochemistry, male, and nonhuman), **Cluster 3** (human.tissue, human. cell, human, adult, stem. cells, middle. aged), **Cluster 4** (genetics, mesenchymal. stroma.cell, metabolism, mesenchymal. stromal.cells, physiology, cytology, drug. effect, humans, adipogenesis, bone. development, gene. expression.regulation, cells, osteogenesis, and cultured), and **Cluster 5** (procedures, upregulation, female, cell. viability, cell. isolation, cell. proliferation, cell. differentiation among others) as shown in Fig. 7a. Similarly, author keywords and keywords in titles were also distributed into five distinguished clusters, as shown in Fig. 7(b) and (c), respectively.

## 4 Discussion

AdMSC is gaining importance in plastic and reconstructive surgery due to its therapeutic applications in fat grafting, facial rejuvenation, scleroderma, and wound healing [16]. The therapeutic potential of AdMSC is mediated by secretory products such as cytokines, growth factors, extracellular vesicles, and proteins [17]. MSCs derived secretomes are gaining importance in ongoing studies since they eliminate the disadvantages of cell-based therapy [3,17,18]. In addition to cytokines, growth factors, and proteins, the MSCs secretomes contain exosomes carrying non-coding RNAs (miR-21, miR-24, and miR-26), further adding to the therapeutic potential of MSCs [17]. The AdMSC-derived secretomes have already exhibited proangiogenic, immunomodulatory, and neurotrophic activities that can be used for managing inflammatory, autoimmune, and neurodegenerative diseases [17,19]. In addition, AdMSC-derived exosomes accelerate wound healing by promoting angiogenesis, fibroblast, or keratinocyte proliferation, regulating inflammatory response, and remodeling of extracellular matrix [20]. The present study provides an overview of global AdMSC research. Understanding the leading journals, institutions, and funding agencies will help prospective researchers design future studies. The emerging trends will further help to decide the direction of their research.

Contrary to Web of Science (WoS), Scopus is available as a single database. In addition, the Scopus database is more accessible to individuals and provides free access to author and source information [12]. Furthermore, the Scopus database offers about 20% more coverage than WoS [21]. This is the primary reason for selecting the Scopus database in our study. The number of studies on AdMSC has increased over the past several years. This indicates the increased awareness among the research community regarding the potential clinical applications of AdMSC.

In a previous bibliometric analysis on AdMSC research that analyzed data between 2003 and 2017 from the PubMed database, the authors categorized the data into three time periods (2003–2007, 2008 to 2012, and 2013 to 2017) for facilitating comparative analysis [10]. As a result, the United States was identified as the most productive country in all three time periods [10]. However, our study identified China to be the most productive country. This disparity can be attributed to the fact that several journals indexed in Scopus are not available in the PubMed database (National Centre for Biotechnology Information) [21].

Even though Biochemical and Biophysical Research Communications, Tissue Engineering, and Stem Cells constituted the top three journals during the 2003 to 2007 period, it was replaced by Tissue Engineering - Part A, Biomaterials, and Stem Cells and Development during 2008–2012 and later by Plos One, Tissue Engineering - Part A, and Plastic and Reconstructive Surgery during 2013–2017 [10]. However, our study identified Stem Cell Research and Therapy (Springer Nature), Plos One (Public Library of Science), and Stem Cells and Development (Mary Ann Liebert) as the top three journals publishing AdMSC research during the 2005 to 2021 period.

Our study identified Seoul National University (South Korea) as the most productive institute/university in AdMSC, with 72 articles published. A similar outcome was reported in another study that evaluated umbilical cord-derived mesenchymal stem cell research from the WoS database in which Seoul National University contributed 48 papers during the 1975 to 2017 period [22]. Similarly, the National Natural Science Foundation of China emerged as the top funding agency in umbilical cord-derived mesenchymal stem cell research [22]. This pattern was also identified in our study, indicating a similar research interest in AdMSC and umbilical cord-derived mesenchymal stem cell research. Furthermore, the National Natural Science Foundation of China (https://www.nsfc.gov.cn/english/site_1/index.html) has always shown an interest in funding research projects in the fields of medicine and plastic surgery [23].

The present study analyses the research trends in the Scopus database and peripherally gives a snapshot of AdMSC research progress. Although it provides data on the research productivity, a few limitations have to be considered. This study was purely based on the Scopus database and did not consider databases such as WoS, PubMed, Google Scholar, etc. Furthermore, false-positive and false-negative results might have slightly affected the result of the bibliometric study. We also did not exclude self-citations during analysis, which may impact the overall number of citations and h-index.

## 5 Conclusions

China was found to be the most productive country, whereas Seoul National University (South Korea) identified as the most productive institute/university in AdMSC research. In addition, the National Natural Science Foundation of China funded the most research studies in AdMSC research. China has a robust national and international collaboration in AdMSC research with the highest single country and multiple country publications. However, the top ten listed countries involved in AdMSC research have a low Multiple Country Publications ratio (MCP Ratio less than 0.50), indicating lower international collaboration. Although Stem Cell Research and Therapy journal published the highest number of articles on AdMSC research, maximum citations were received by Stem Cells and Development journal. The findings from this study indicate a progressive increase in interest among the research community towards AdMSC, suggesting promising prospects in the coming years. Furthermore, our study provides a fresh perspective of global AdMSC research, enabling us to understand the past, present, and future.

## Funding

No substantial funding is to be stated.

## Availability of data and material

The data that support the findings of this study are available in the form of supplementary material attached to the publication.

## Ethics approval

Ethics committee approval is not required as there is no human or animal research.

## Consent to participate

Not applicable.

## Consent for publication

Not applicable.

## Code availability

Not applicable.

## Author contribution

KS and THM was involved in the conception and design of the study, collected the data, performed the analysis, interpretation, and wrote the initial draft. HHM, RK, AMP, VC, HST, KD, Amarpal, and GTS participated in the study and analysis. KS, GTS, and Amarpal critically revised the manuscript. All authors certify that they have made a direct and substantial contribution to the work reported in the manuscript and have approved the final version of the manuscript.

## Declaration of competing interest

All authors declare that there exist no commercial or financial relationships that could, in any way, lead to a potential conflict of interest.
